# Psychological Well-Being and Innovative Work Behavior Among ICT Employees in Türkiye: Comparing the Mediating Roles of Creative Self-Efficacy and Subjective Vitality

**DOI:** 10.3390/bs16081433

**Published:** 2026-08-20

**Authors:** Nagihan Kartal, Fazlı Yıldırım, Mustafa Aslan

**Affiliations:** 1 Faculty of Economics, Administration, and Social Sciences, Istanbul Topkapi University, 34087 Istanbul, Türkiye; 2 Faculty of Computer and Information Sciences, Yeditepe University, 34755 Istanbul, Türkiye; fazli.yildirim@yeditepe.edu.tr; 3 Faculty of Applied Sciences, Istanbul Bilgi University, 34060 Istanbul, Türkiye; mustafa.aslan@bilgi.edu.tr

**Keywords:** innovative work behavior, psychological well-being, creative self-efficacy, subjective vitality, ICT sector, PLS-SEM

## Abstract

Innovation is an everyday imperative in the information and communication technology (ICT) sector, yet the psychological conditions through which well-being is associated with innovative action remain insufficiently understood. This study investigates whether subjective vitality and creative self-efficacy constitute distinct psychological pathways—an energy-based and a belief-based mechanism, respectively—through which psychological well-being becomes associated with innovative work behavior, thereby providing a more differentiated examination of the assumption that psychologically healthy employees are more likely to behave innovatively. An online survey was administered to 463 ICT-sector employees in Türkiye, and partial least squares structural equation modeling (PLS-SEM) was used to test direct and indirect relationships with comprehensive assessment of measurement quality, predictive relevance, and effect sizes. Creative self-efficacy emerged as a meaningful indirect pathway linking psychological well-being to innovative work behavior, whereas subjective vitality, though strongly predicted by well-being, showed no significant relationship with innovative outcomes. These findings suggest that not all positive psychological states are equally innovation-relevant: among ICT employees, the indirect association between well-being and innovation was evident through employees’ confidence in their own creative capability rather than through feelings of psychological energy—a distinction with meaningful implications for both theory and practice in knowledge-intensive, digitally dynamic organizations.

## 1. Introduction

In contemporary organizational environments characterized by digital transformation, continuous uncertainty, and intensifying innovation pressure, employees are expected not merely to perform assigned tasks but to proactively generate, champion, and implement novel ideas (Anderson et al., 2014; Yuan & Woodman, 2010). In the ICT sector specifically, where technological shifts emerge rapidly and competitive advantages quickly become obsolete, innovative work behavior has evolved from a desirable competency into a strategic necessity. Yet despite this growing emphasis, organizations continue to struggle with a fundamental question: why do some psychologically healthy employees actively engage in innovative behavior while others—operating within identical organizational environments—remain passive?

Yet the intuitive expectation that psychologically healthy or energized employees will naturally exhibit greater innovative behavior conflates two conceptually distinct processes. Innovative work behavior involves not only motivational activation but also uncertainty, risk-taking, challenging established routines, and defending unconventional ideas within organizational contexts that may resist change (Scott & Bruce, 1994). Consequently, employees may require not merely vitality and motivation, but also confidence in their own creative capability—a specific form of psychological agency that generalized energy alone cannot provide.

Drawing upon self-determination theory (Ryan & Deci, 2000) and social cognitive theory (Bandura, 1997), the present study proposes that psychological well-being may be associated with innovative work behavior through at least two theoretically distinct pathways: an energy-based pathway represented by subjective vitality, and a belief-based pathway represented by creative self-efficacy. While vitality reflects feelings of aliveness and psychological energy, creative self-efficacy reflects employees’ confidence in their capacity to generate creative outcomes and engage in innovation-related tasks. Critically, the present study argues that these pathways are not equivalent in their innovation relevance—and that the belief-based pathway may constitute the more proximal psychological mechanism for translating well-being into innovative action.

These two theories are employed not as parallel, disconnected rationales but as complementary lenses on a single resource-conversion process. Self-determination theory describes how psychological well-being is linked to broad motivational resources: when basic psychological needs are satisfied, individuals experience heightened intrinsic motivation and psychological energy (Ryan & Deci, 2000; Ryan & Frederick, 1997). Social cognitive theory, in turn, explains how such resources are—or are not—converted into domain-specific action: behavior follows not from available energy per se, but from efficacy beliefs that govern behavioral initiation, effort investment, and persistence (Bandura, 1997).

In the integrated framework adopted here, social cognitive theory serves as the primary explanatory foundation for innovation-directed behavior, while self-determination theory provides the supporting account of the energetic resources that well-being makes available.

This distinction carries meaningful theoretical implications. Prior research linking psychological well-being and related positive states to innovative work behavior has, in fact, examined a diverse set of intervening psychological mechanisms rather than assuming a direct link. Creative self-efficacy has been established as one of the most robust individual-level predictors and mediators of creative and innovative behavior (Tierney & Farmer, 2002; Newman et al., 2018; Ng & Lucianetti, 2016), whereas energy-related states such as vitality, aliveness, and thriving have been linked primarily to creative work involvement and engagement-related outcomes (Kark & Carmeli, 2009; Carmeli & Spreitzer, 2009). Other streams have emphasized motivational, empowerment-based, and job-design mechanisms in the emergence of employee creativity and innovation (Anderson et al., 2014; Yuan & Woodman, 2010; Liu et al., 2017).

What remains largely absent from the literature, however, is a direct comparison: studies that estimate an energy-based mechanism (subjective vitality) and a belief-based mechanism (creative self-efficacy) simultaneously within the same structural model, so that their relative innovation relevance can be evaluated against each other rather than in isolation.

A recent partial exception is Li et al. (2025), who modeled teaching efficacy and work vitality as parallel and sequential mediators between teachers’ emotional competence and innovative teaching behaviors in a sample of school teachers. That study, however, examined general teaching efficacy rather than domain-specific creative self-efficacy, operationalized energy through the vitality subdimension of thriving rather than subjective vitality in the self-determination tradition, and did not position the two mechanisms as competing theoretical pathways.

To the best of our knowledge, no prior study has comparatively tested subjective vitality and creative self-efficacy as simultaneous mechanisms linking psychological well-being to innovative work behavior, and none has done so among ICT employees; the incremental contribution of the present study accordingly lies in its focal constructs, its antecedent, its explicitly comparative framing, and its sector.

The central contribution of this study lies in directly comparing two theoretically distinct indirect pathways within the same structural model: an energy-based pathway represented by subjective vitality and a belief-based pathway represented by creative self-efficacy. By estimating these pathways simultaneously, the study clarifies their relative associations with innovative work behavior among ICT employees and extends existing evidence on the psychological mechanisms linking well-being and innovation.

The ICT context provides reasons to expect that the association between vitality and innovative work behavior may be attenuated. Two plausible explanations are considered. The first is a range-restriction account, under which limited variation in vitality reduces its ability to differentiate employees. The second is a resource-conversion account, under which vitality may be less closely associated with innovative behavior than domain-specific creative self-efficacy. These explanations are revisited cautiously in light of the results.

The study addresses the following research questions:

RQ1. Is psychological well-being significantly associated with innovative work behavior?
RQ2. Do creative self-efficacy and subjective vitality function as distinct psychological mechanisms in the relationship between psychological well-being and innovative work behavior?
RQ3. Do creative self-efficacy and subjective vitality differ in their roles as psychological mechanisms linking psychological well-being to innovative work behavior?

## 2. Literature Review

### 2.1. Psychological Well-Being and Innovative Work Behavior

Psychological well-being (PWB) is a multidimensional construct of positive psychological functioning that encompasses self-acceptance, environmental mastery, autonomy, positive relations, purpose in life, and personal growth (Ryff, 1989, 2014). Far from signifying the mere absence of distress, PWB reflects individuals’ capacity to flourish, realize their potential, and engage meaningfully with work and social life. In organizational settings, PWB has been associated with resilience, intrinsic motivation, and proactive behavior (Luthans et al., 2006; Wright & Cropanzano, 2004).

Conceptually, the present study adopts a eudaimonic perspective on well-being, in the tradition of Ryff’s (1989, 2014) positive-functioning framework, rather than a hedonic (subjective well-being) perspective centered on affect balance and life satisfaction. Operationally, PWB was assessed with the flourishing-based measure of Diener et al. (2009), which captures self-perceived positive functioning across relationships, purpose, engagement, competence, and self-regard, and is widely used as a parsimonious indicator of eudaimonic functioning. Throughout this study, PWB therefore refers to positive psychological functioning in this eudaimonic sense.

Beyond its association with general positive functioning, PWB may be particularly relevant in innovation-oriented contexts where employees are expected to cope with ambiguity, continuously adapt to change, and proactively generate novel solutions. Employees with higher PWB tend to demonstrate psychological flexibility, cognitive openness, and stronger intrinsic motivation—psychological qualities that facilitate engagement with uncertain and challenging tasks (Amabile, 1996; Fredrickson, 2001). From the perspective of broaden-and-build theory, positive psychological functioning may broaden individuals’ cognitive repertoires and increase their willingness to explore unconventional ideas (Fredrickson, 2001).

Importantly, however, the assumption that psychologically healthy employees naturally become innovative employees deserves closer theoretical scrutiny. Innovative work behavior differs from general positive work behaviors because it involves greater uncertainty, experimentation, and creative risk-taking (Anderson et al., 2014). Employees with strong PWB may possess the psychological resources necessary for engaging in innovation-related activities, yet whether these resources reliably translate into innovative action may depend on additional psychological mechanisms—particularly those involving cognitively grounded creative confidence.

On the basis of this reasoning, the following hypothesis is proposed:

**H1.**  *Psychological well-being is positively associated with innovative work behavior.*

### 2.2. Psychological Well-Being and Subjective Vitality

Subjective vitality (SV) refers to the dynamic experience of aliveness, energy, and vigor—reflecting one’s felt sense of motivational readiness and psychological liveliness (Ryan & Frederick, 1997). According to self-determination theory, vitality emerges as a key outcome of basic psychological need fulfillment: when individuals’ needs for autonomy, competence, and relatedness are satisfied, they experience heightened intrinsic motivation and psychological energy (Ryan & Deci, 2000). Employees who report high PWB are consequently more likely to experience motivational activation and vitality at work (Kark & Carmeli, 2009).

Subjective vitality is more than a transient affective state; it reflects a psychologically energized condition that enables employees to approach work with enthusiasm, sustain engagement during demanding tasks, and maintain proactive functioning even under organizational pressures. Prior research has established consistent positive associations between PWB and workplace vitality across diverse organizational samples (Carmeli & Spreitzer, 2009). In ICT environments characterized by continuous cognitive demands and rapid change, this energized state may be particularly relevant for maintaining adaptive functioning.

**H2.**  *Psychological well-being is positively associated with subjective vitality.*

### 2.3. Psychological Well-Being and Creative Self-Efficacy

Creative self-efficacy (CSE) is a domain-specific form of efficacy belief reflecting individuals’ confidence in their ability to generate original and useful ideas and to successfully engage in innovation-related tasks (Tierney & Farmer, 2002). Grounded in social cognitive theory (Bandura, 1997), CSE captures the conviction that one can overcome obstacles to develop and implement novel solutions. Employees who experience greater PWB are more likely to perceive themselves as competent, autonomous, and psychologically resilient—attributes that strengthen creative self-beliefs (Wright & Cropanzano, 2004).

Psychological well-being may contribute to CSE through multiple mechanisms. First, employees with higher PWB may interpret work challenges as manageable opportunities rather than threats, thereby reinforcing their willingness to engage in creative experimentation. Second, the sense of personal agency and self-worth associated with PWB may directly strengthen domain-specific beliefs regarding creative capability. In ICT environments that continuously require adaptive and innovative responses to emerging technological challenges, such creative self-beliefs may serve as an important psychological foundation for innovation-related action.

**H3.**  *Psychological well-being is positively associated with creative self-efficacy.*

### 2.4. Subjective Vitality and Innovative Work Behavior

Subjective vitality provides the motivational energy and persistence necessary for sustained work engagement and proactive functioning (Ryan & Frederick, 1997). Employees who feel vitalized are generally more willing to invest cognitive and emotional effort in demanding tasks, more resilient in the face of obstacles, and more proactive in approaching challenging work situations (Kark & Carmeli, 2009). From this perspective, vitality may support innovative work by sustaining the psychological energy required for idea generation, advocacy, and implementation.

Two more specific mechanisms can be articulated. At the cognitive level, energized psychological states may broaden attentional focus and thought–action repertoires, facilitating the exploratory and associative thinking from which novel ideas emerge (Fredrickson, 2001). At the behavioral level, vitality may supply the persistence needed to champion ideas and carry them through implementation despite obstacles and setbacks (Kark & Carmeli, 2009). Consistent with this reasoning, feelings of vitality and aliveness have been positively associated with creative work involvement (Kark & Carmeli, 2009), and thriving-related states have been linked to innovative behaviors at work (Carmeli & Spreitzer, 2009).

However, the relationship between vitality and innovative work behavior is theoretically less straightforward than its relationship with engagement or thriving. Innovation differs from general work engagement because it requires not only persistent effort but also uncertainty tolerance, willingness to challenge established routines, and readiness to defend unconventional ideas against organizational resistance (Anderson et al., 2014). Employees may feel psychologically energized while still avoiding innovative action if they perceive creative efforts as risky or doubt their creative competence. Although prior theory supports a positive association between subjective vitality and innovative work behavior, the ICT context provides reasons to expect that this association may be attenuated. Vitality may have limited discriminatory power if its variance is restricted, or it may be less closely associated with innovative behavior than domain-specific creative self-efficacy. H4 nevertheless retains the established positive theoretical expectation, while these contextual alternatives are revisited in the Discussion.

**H4.**  *Subjective vitality is positively associated with innovative work behavior.*

### 2.5. Creative Self-Efficacy and Innovative Work Behavior

Creative self-efficacy has emerged as one of the most robust psychological predictors of innovative work behavior across organizational settings (Tierney & Farmer, 2002; Newman et al., 2018). Unlike generalized motivational states, CSE specifically captures employees’ perceived capability to perform creatively under uncertain and challenging conditions. Employees with stronger creative self-beliefs are more likely to initiate innovative actions, persist despite setbacks, continue advocating for their ideas when facing organizational resistance, and invest sustained effort in innovation-related activities.

Social cognitive theory explains these relationships through the mechanisms of behavioral initiation, effort investment, and persistence under difficulty (Bandura, 1997). In innovation contexts, these mechanisms are particularly relevant because innovative behavior frequently involves ambiguity, experimentation, and social risk-taking. Employees who lack confidence in their creative ability may avoid proposing unconventional ideas even when they possess sufficient knowledge or motivational energy. Conversely, employees with strong CSE may be more willing to challenge routines, engage in creative problem-solving, and take ownership of innovation-related initiatives—behaviors that are especially valued in ICT environments where continuous novel problem-solving is organizationally expected.

**H5.**  *Creative self-efficacy is positively associated with innovative work behavior.*

### 2.6. Mediation Effects of Subjective Vitality and Creative Self-Efficacy

Before the mediation hypotheses are developed, the selection of these two mechanisms over other candidate mediators warrants explicit justification. Constructs such as work engagement, thriving at work, psychological empowerment, intrinsic motivation, and proactive motivation have all been linked to innovative outcomes and are conceptually adjacent to innovative behavior. However, each of these constructs blends energetic and cognitive–evaluative components to varying degrees: thriving combines vitality with learning, empowerment combines competence with meaning and impact, and engagement combines vigor with dedication and absorption.

Subjective vitality and creative self-efficacy were selected precisely because they constitute comparatively pure exemplars of the two theoretical pathways under comparison. Vitality is the most direct operationalization of felt psychological energy within self-determination theory (Ryan & Frederick, 1997), whereas creative self-efficacy is the most domain-specific operationalization of creative agency within social cognitive theory (Bandura, 1997; Tierney & Farmer, 2002). Testing composite constructs would confound the energy-versus-belief comparison at the heart of this study; testing these two focal constructs keeps the comparison theoretically interpretable.

Psychological well-being may be associated with innovative behavior through more specific psychological mechanisms that facilitate creative action.

The present study proposes two such mechanisms operating in parallel: an energy-based pathway via subjective vitality, and a belief-based pathway via creative self-efficacy.

The energy-based pathway suggests that employees who experience PWB feel more psychologically activated and motivated, which may increase their persistence, proactive engagement, and willingness to invest effort in innovation-oriented tasks. The belief-based pathway suggests that PWB is positively associated with employees’ perceptions of competence and agency, which in turn enhances their confidence in their own creative capability. Employees who perceive themselves as creatively capable may then be more likely to generate, advocate, and implement innovative ideas despite uncertainty or organizational resistance.

Critically, the present study argues that these pathways are not equivalent. Drawing upon social cognitive theory, we contend that creative self-efficacy—as a cognitively grounded, domain-specific belief—may constitute a more proximal and innovation-relevant mechanism than subjective vitality. Innovation involves not only energy and persistence but identity-based confidence: the belief that one is capable of contributing creatively within an uncertain organizational context. Employees may feel energized yet still conform behaviorally if they lack this creative confidence. In ICT environments, creative self-efficacy may therefore represent a more proximal psychological correlate of innovative behavior than subjective vitality. The salience of this belief-based resource is likely to grow as ICT work becomes increasingly AI-assisted: experimental evidence indicates that intelligent AI assistants raise employees’ innovation behavior through gains in creative self-efficacy (Yin et al., 2024), and field evidence shows that AI assistance can augment employee creativity (Jia et al., 2024).

**H6.**  *Subjective vitality is expected to play a mediating role in the relationship between psychological well-being and innovative work behavior.*

**H7.**  *Creative self-efficacy is expected to play a mediating role in the relationship between psychological well-being and innovative work behavior.*

**Figure 1:**
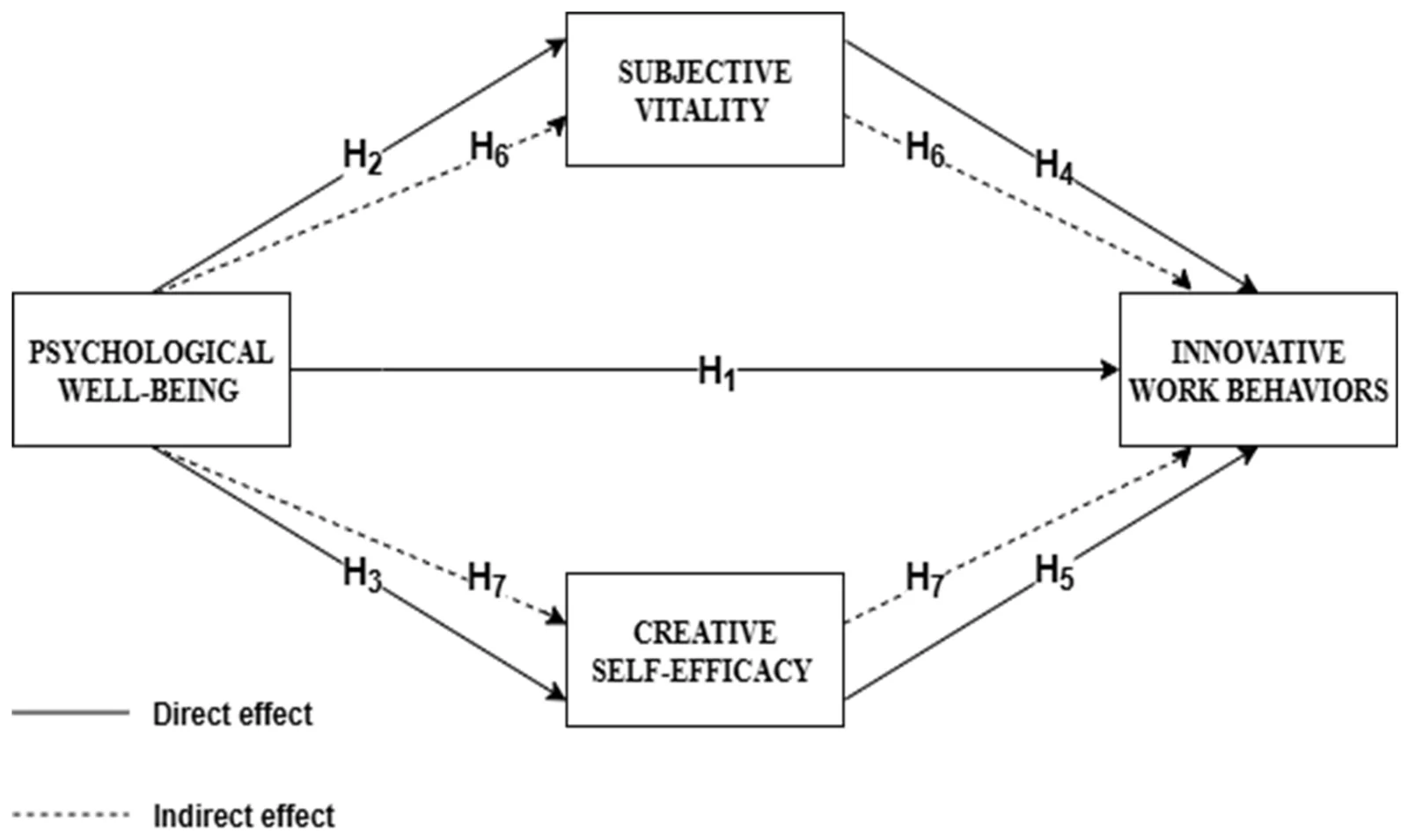
Conceptual Model.

Figure 1 presents the conceptual model based on the theoretical framework and hypotheses developed above.

## 3. Methods

### 3.1. Participants and Procedure

This cross-sectional study adopted an online survey method to collect data from employees in Türkiye’s ICT sector. Data were collected between 20 April 2025 and 21 June 2025. A convenience sampling approach was adopted; participants were recruited via e-mail and LinkedIn invitations.

Convenience sampling was employed because no publicly accessible sampling frame of ICT-sector employees exists in Türkiye, rendering probability-based sampling infeasible; professional networks and organizational e-mail lists provided the most direct access to the target population. Invitations contained a brief description of the study’s purpose and a link to the online questionnaire.

Eligibility was restricted to individuals currently employed in the ICT sector: prospective participants identified via LinkedIn were screened prior to invitation by inspecting their publicly available profile information (current employer and sector), and e-mail invitations were likewise directed to employees of ICT-sector organizations. In addition, the questionnaire included demographic items on current position, sector of employment, tenure in the current position, and total years of work experience, and responses to these items were used to verify sector membership; respondents employed outside the ICT sector were treated as out-of-scope and excluded.

Because invitations were disseminated across multiple channels over an extended period and the number of individuals ultimately reached could not be reliably tracked, a precise response rate could not be computed. The potential selection bias associated with LinkedIn-based recruitment, particularly the possible over-representation of more highly educated and professionally networked employees, is acknowledged as a limitation of the study. After removing incomplete or out-of-scope responses, the final sample consisted of a total of 463 employees, where 386 (83.4%) of them were male and 77 (16.6%) females.

Most of the participants were between 26 and 33 years of age (44.7%), and held at least a bachelor’s degree (53.3%). Respondents reported varying levels of organizational tenure, with majority reporting more than nine years of total work experience. The demographics of the participants are given in Table 1.

During data collection, several procedural remedies were applied to reduce the risk of common method bias (Podsakoff et al., 2003). Participants were informed that their responses would remain strictly anonymous and would be used exclusively for research purposes. They were encouraged to respond honestly and were explicitly told that there were no correct or incorrect answers. The questionnaire employed previously validated scales with clear and standardized instructions, and research variables were presented in a consistent format to minimize evaluation anxiety and social desirability bias.

### 3.2. Measures

All constructs were measured using previously validated scales adapted to Turkish, with a five-point Likert-type response format ranging from 1 (strongly disagree) to 5 (strongly agree). For each instrument, the Cronbach’s alpha reported below refers to the original validation study. Representative sample items are presented below. The items were administered in Turkish; the original English wording is provided for readers’ convenience.

The Psychological Well-Being (PWB) was assessed by using the 8-item scale developed by Diener et al. (2009) and adapted to Turkish by Telef (2013). The original scale reported a Cronbach’s alpha of 0.80.

Two sample items are as follows:

- *I lead a purposeful and meaningful life.*
- *My social relationships are supportive and rewarding.*

The Subjective Vitality (SV) construct was measured by using the 7-item scale developed by Ryan and Frederick (1997) and adapted to Turkish by Uysal et al. (2013). The original scale has one reverse-coded item (item 2) and the Cronbach’s alpha of the original scale is 0.84.

Two sample items are:

- *I feel alive and vital.*
- *I don’t feel very energetic* (reverse-coded item).

The Creative Self-Efficacy (CSE) was measured by using the 8-item scale developed by Carmeli and Schaubroeck (2007) and adapted to Turkish by Alayoğlu (2019). The Cronbach’s Alpha of the original scale is 0.92.

Two sample items from this scale are:

- *I will be able to achieve most of the goals that I have set for myself in a creative way.*
- *When facing difficult tasks, I am certain that I will accomplish them creatively.*

The Innovative Work Behavior (IWB) was assessed using the 6-item scale developed by Scott and Bruce (1994) and adapted to Turkish by Çalışkan et al. (2019). The Cronbach’s alpha of the original scale is 0.93, and two sample items are:

- *I generate creative ideas.*
- *I develop plans and schedules for implementing new ideas.*

### 3.3. Analytic Strategy

The proposed research model was evaluated using partial least squares structural equation modeling (PLS-SEM) with SmartPLS 4.0 (Ringle et al., 2015). The selection of PLS-SEM over covariance-based SEM (CB-SEM) was justified on both theoretical and analytical grounds.

First, the research objective is explanatory-predictive—combining explanation with prediction—rather than strictly confirmatory. Beyond testing whether the hypothesized paths are statistically significant, the study aims to compare the relative explanatory and predictive contributions of two mediating mechanisms to innovative work behavior. PLS-SEM is specifically recommended when the analytical goal involves identifying and comparing the relative importance of driver constructs for a focal outcome and when out-of-sample predictive assessment forms part of the model evaluation, whereas CB-SEM is preferable when the objective is strict theory confirmation through global fit assessment (Hair et al., 2019; Sarstedt et al., 2022).

Second, the model includes two parallel mediating variables estimated simultaneously within a single structural framework, which is analytically well-suited to PLS-SEM’s iterative estimation approach.

Third, PLS-SEM performs reliably under the reflective measurement specifications employed in this study and does not impose multivariate normality assumptions.

Fourth, PLS-SEM has been widely adopted and validated in organizational psychology research with moderately sized samples and complex mediation structures (Hair et al., 2019, 2022).

The adequacy of the sample size was evaluated using the inverse square root method (Kock & Hadaya, 2018), following the guidelines presented by Hair et al. (2021, p. 17): assuming a statistical power of 80%, a significance level of 5%, and a minimum path coefficient (p_min_) of 0.20, the minimum required sample size is n_min_ > (2.486/0.20)^2^ = 154.505, which rounds to 155 observations. The achieved sample of 463 comfortably exceeds this requirement, as well as more conservative rules of thumb for models with a maximum of three structural predictors per endogenous construct.

Model evaluation followed a two-stage procedure. In the first stage, the measurement model was assessed for internal consistency (Cronbach’s alpha, Composite Reliability—CR), convergent validity (Average Variance Extracted—AVE ≥ 0.50), and discriminant validity using the heterotrait–monotrait ratio (HTMT) with bootstrapped 95% confidence intervals (Henseler et al., 2015).

Multicollinearity was evaluated using variance inflation factor (VIF) values. In the second stage, the structural model was assessed through path coefficients (β), bootstrapped t-statistics (5000 resamples), explained variance (R^2^), effect sizes (f^2^; Cohen, 1988), and predictive relevance (Q^2^) via the blindfolding procedure (omission distance = 7).

Common method bias was evaluated through both procedural and statistical remedies. Full collinearity VIF values for all constructs remained below 3.3 (Kock, 2015), and Harman’s single-factor test indicated that the first unrotated factor explained 31.7% of the total variance, well below the 50% threshold, confirming that no single factor accounted for the majority of the variance. To assess non-response bias, early (first 25%) and late (last 25%) respondents were compared using independent-samples *t*-tests across all four constructs; no significant differences emerged (all *p* > 0.05), suggesting non-response bias is not a concern.

## 4. Results

### 4.1. Measurement Model: Reliability, Validity, and Factor Loadings

Table 2 presents the full measurement model results, including factor loadings for all scale items, internal consistency coefficients, composite reliability, and average variance extracted. All reliability and validity statistics reported in Table 2 were computed from the present sample. Cronbach’s alpha coefficients ranged from 0.870 (PWB) to 0.937 (CSE), indicating acceptable to excellent internal consistency across all constructs. Reliability was additionally assessed using Dijkstra–Henseler’s ρA, which ranged from 0.872 (PWB) to 0.937 (CSE), with all values exceeding the recommended 0.70 threshold (Hair et al., 2019). Composite reliability (CR) values ranged from 0.898 to 0.948, exceeding the recommended threshold of 0.70. Average variance extracted (AVE) values ranged from 0.523 to 0.694, all surpassing the 0.50 threshold required for convergent validity (Hair et al., 2019).

Individual indicator loadings were generally acceptable across the constructs (see Table 2). Four indicators had loadings below the conventional 0.708 benchmark: PWB07 (λ = 0.680), IWB03 (λ = 0.559), SV02 (λ = 0.497), and SV03 (λ = 0.612). Following Hair et al. (2021, pp. 76–77), indicators with loadings between 0.40 and 0.708 should be considered for removal only when their deletion leads to meaningful improvements in composite reliability or average variance extracted and when content validity is not compromised; only loadings below 0.40 warrant automatic elimination.

In the present study, all constructs already exceeded the recommended thresholds for composite reliability and AVE, and removal of the lowest-loading indicators produced negligible changes in AVE (<0.02) and CR (<0.01); accordingly, no indicator met the removal criterion. All indicators were therefore retained in the final measurement and structural models to preserve the content validity and comparability of the original validated instruments.

Content-validity considerations were particularly relevant for the two lowest-loading indicators. SV02 is the sole reverse-scored item of the subjective vitality scale; its removal would eliminate the instrument’s only safeguard against acquiescent response patterns and would narrow the conceptual breadth of the construct, and attenuated loadings are a well-documented psychometric characteristic of reverse-scored items rather than evidence of construct irrelevance.

IWB03 (“I support and encourage other employees’ ideas”) was retained because it captures the idea-promotion facet of innovative work behavior, reflecting employees’ efforts to support, advocate, and encourage innovative ideas within the workplace. Although its loading was lower than those of the other indicators, the item represents a conceptually distinct aspect of innovative behavior that is not fully captured by the remaining items. Its retention therefore helps preserve the content validity and conceptual breadth of the original scale.

As an additional sensitivity analysis, an alternative structural model was estimated after temporarily excluding the two indicators with the lowest loadings, IWB03 and SV02. This alternative specification was conducted solely to assess whether the retention of these indicators affected the substantive findings; it was not used as the final model. All path coefficients retained their original signs and significance levels, and the largest change in any coefficient was 0.022. The substantive conclusions were therefore unchanged. On this basis, the final reported model retained all indicators, including IWB03 and SV02. Specifically, the alternative model produced the following estimates: PWB–CSE: β = 0.510, t = 9.242; PWB–SV: β = 0.661, t = 21.675; PWB–IWB: β = 0.203, t = 3.661; CSE–IWB: β = 0.513, t = 8.267, all *p* < 0.001; and SV–IWB: β = 0.064, t = 1.033, *p* = 0.301. All AVE values remained above 0.50 (CSE = 0.694; IWB = 0.699; PWB = 0.523; SV = 0.654), and all reliability coefficients remained within acceptable ranges.

### 4.2. Discriminant Validity and Collinearity

Discriminant validity was assessed using the HTMT ratio with bootstrapped 95% confidence intervals (Table 3). All HTMT point estimates were below the recommended threshold of 0.90 (range: 0.427–0.729). Bootstrapped confidence intervals confirmed that no upper bound approached or exceeded this threshold, providing robust evidence for the discriminant validity of all constructs (Henseler et al., 2015).

Collinearity was assessed using variance inflation factor (VIF) values. As shown in Table 4, all VIF values remained well below the threshold of 5.0 (range: 1.365–2.016), indicating that multicollinearity did not constitute a concern in the structural model.

Full collinearity VIF values were additionally computed for all constructs as a CMV sensitivity check (Kock, 2015); all values remained below 3.3, providing no indication of severe common method variance.

### 4.3. Explained Variance and Predictive Relevance

Table 5 reports the R^2^ and Q^2^ values for all endogenous constructs. The model demonstrated substantial explanatory power: psychological well-being explained 42.9% of the variance in subjective vitality (R^2^ = 0.429) and 26.1% of the variance in creative self-efficacy (R^2^ = 0.261). Together, PWB and the two mediating variables accounted for 46.2% of the variance in innovative work behavior (R^2^ = 0.462), indicating moderate explanatory power.

Stone–Geisser Q^2^ values obtained through the blindfolding procedure (omission distance = 7) exceeded zero for all endogenous constructs (CSE: Q^2^ = 0.173; IWB: Q^2^ = 0.280; SV: Q^2^ = 0.249), indicating adequate predictive relevance (Hair et al., 2019).

Out-of-sample predictive performance was further assessed using the PLSpredict procedure (Shmueli et al., 2019; Hair et al., 2021). All indicators yielded positive Q^2^ predict values, with the indicators of the key target construct, innovative work behavior, ranging from 0.103 to 0.191, indicating that the model outperforms the naive mean-value benchmark. When prediction errors were compared against the linear regression model (LM) benchmark, the PLS-SEM model produced lower RMSE values for five of the six IWB indicators, indicating medium predictive power according to the guidelines of Shmueli et al. (2019).

### 4.4. Structural Model Results: Direct Effects

**Figure 2:**
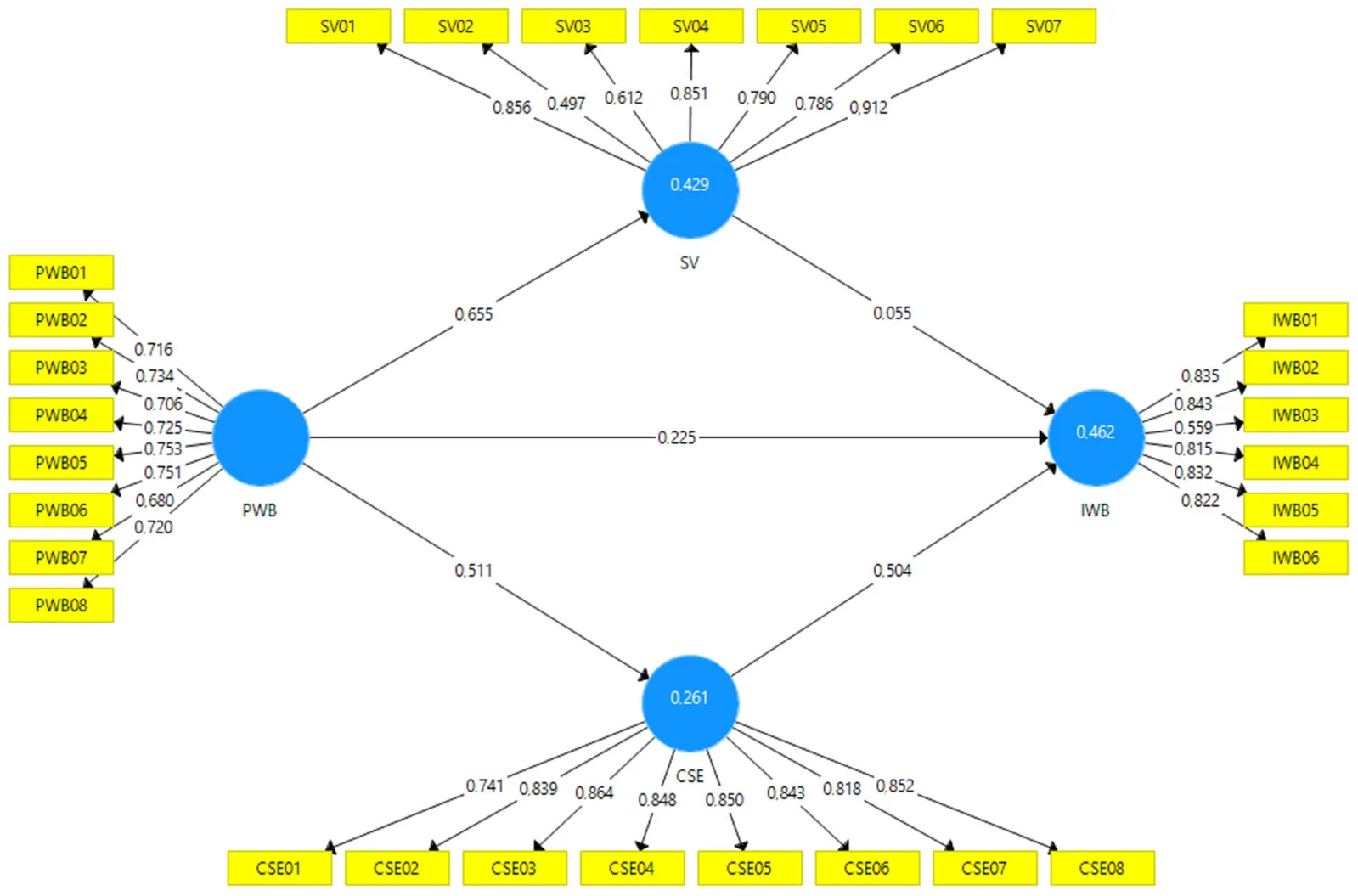
Structural model.

Following the conceptual model (see Figure 1), the structural model was specified and estimated in SmartPLS 4.0 using 5000 bootstrap resamples to obtain robust path coefficient estimates and significance levels. Figure 2 presents the resulting structural model with standardized path coefficients and explained variance values for all endogenous constructs. The model explains 42.9% of the variance in subjective vitality, 26.1% in creative self-efficacy, and 46.2% in innovative work behavior, reflecting moderate overall explanatory power.

Analysis results (the direct path coefficients with bootstrapped standard errors, t-statistics, *p*-values, and 95% bias-corrected bootstrap confidence intervals) are reported in Table 6. The Cohen’s f^2^ effect sizes were also calculated and interpreted as 0.02 ≤ f^2^ < 0.15 as small, 0.15 ≤ f^2^ < 0.35 as medium, and f^2^ ≥ 0.35 as large. As per the analysis results, psychological well-being was significantly associated with creative self-efficacy (β = 0.511, SE = 0.056, t = 9.130, *p* < 0.001, f^2^ = 0.353; H3 supported), subjective vitality (β = 0.655, SE = 0.030, t = 21.509, *p* < 0.001, f^2^ = 0.751; H2 supported), and innovative work behavior (β = 0.225, SE = 0.056, t = 3.986, *p* < 0.001, f^2^ = 0.047; H1 supported). Creative self-efficacy was also significantly associated with innovative work behavior (β = 0.504, SE = 0.062, t = 8.155, *p* < 0.001, f^2^ = 0.344; H5 supported). In contrast, the path from subjective vitality to innovative work behavior was not statistically significant (β = 0.055, SE = 0.062, t = 0.894, *p* = 0.371; H4 is not supported), and the effect size was negligible (f^2^ = 0.003).

The effect size estimates clarify the practical significance of association between PWB and SV (f^2^ = 0.751), confirming that well-being is strongly associated with vitality. The SV, however, yielded a negligible effect size (f^2^ = 0.003), indicating that the association between SV and IWB is statistically negligible. By contrast, the PWB–CSE relationship demonstrated a large effect (f^2^ = 0.353), whereas the CSE–IWB relationship demonstrated a medium effect that was close to the conventional large-effect threshold (f^2^ = 0.344), consistently supporting the practical significance of the belief-based pathway.

The indirect effects (see the last two rows of Table 6) show that PWB to IWB through CSE was statistically significant (β = 0.258, SE = 0.046, t = 5.568, *p* < 0.001, 95% CI [0.170, 0.349]), indicating that creative self-efficacy represents a statistically significant indirect pathway in the association between psychological well-being and innovative work behavior (H7 supported).

The indirect path through SV was not significant (β = 0.036, SE = 0.041, t = 0.883, *p* = 0.377, 95% CI [−0.037, 0.122]), indicating that subjective vitality did not exhibit a meaningful indirect effect (H6 not supported).

To provide descriptive context for the non-significant vitality path, the distributional properties of the study variables were examined. Subjective vitality showed the lowest mean among the study constructs (M = 3.60, SD = 0.70) and an approximately symmetric distribution (skewness = −0.33, kurtosis = 0.28). By contrast, the remaining constructs displayed higher means and markedly stronger negative skew (PWB: M = 4.02, SD = 0.56, skewness = −0.90, kurtosis = 3.89; CSE: M = 4.15, SD = 0.58, skewness = −0.84, kurtosis = 3.42; IWB: M = 4.32, SD = 0.52, skewness = −1.41, kurtosis = 6.68). These distributional properties indicate that the non-significant vitality path cannot be attributed to a pronounced ceiling effect or severe range restriction in vitality scores within the present sample: vitality was, in fact, the least skewed construct in the model and exhibited the largest standard deviation among the study variables. The interpretation of this finding is therefore developed in the Discussion with an emphasis on the conversion of energetic resources into innovation-relevant beliefs rather than on within-sample variance restriction.

The significance of one indirect effect and the non-significance of the other do not, by themselves, establish that the two indirect effects differ significantly from each other (Hayes, 2022). Therefore, a formal bootstrap comparison of the two specific indirect effects was conducted. Specifically, within each of the 5000 bootstrap samples, the difference between the two specific indirect effects was calculated by subtracting the subjective-vitality indirect effect from the creative-self-efficacy indirect effect. The indirect effect through creative self-efficacy was significantly larger than the indirect effect through subjective vitality (Δβ = 0.221, 95% bootstrap CI [0.065, 0.368], *p* = 0.006). These findings provide formal statistical evidence that the indirect effect through creative self-efficacy exceeded that through subjective vitality in the present sample.

Finally, given the predominantly male composition of the sample, a sensitivity analysis was conducted by re-estimating the structural model with gender included as a control variable on innovative work behavior. Gender was not significantly associated with innovative work behavior (β = −0.009, t = 0.283, *p* = 0.777), and all hypothesized path coefficients remained substantively unchanged in sign, magnitude, and significance when gender was included (PWB–CSE: β = 0.511; PWB–SV: β = 0.655; PWB–IWB: β = 0.226; CSE–IWB: β = 0.504, all *p* < 0.001; SV–IWB: β = 0.055, *p* = 0.378), indicating that the findings are robust to the gender composition of the sample.

## 5. Discussion

This study compared an energy-based pathway through subjective vitality with a belief-based pathway through creative self-efficacy in the association between psychological well-being and innovative work behavior. The results showed a clear difference between the two pathways: creative self-efficacy was meaningfully associated with innovative work behavior, whereas subjective vitality was not. The discussion therefore focuses on why domain-specific creative confidence may have been more closely associated with innovative action than generalized psychological energy in the present ICT sample.

### 5.1. Psychological Well-Being as a Psychological Foundation for Innovation

The positive association between PWB and IWB (H1 supported) is consistent with prior evidence linking positive psychological functioning to proactive and creativity-related outcomes (Fredrickson, 2001). Employees who report greater purpose, competence, and interpersonal connectedness may demonstrate greater cognitive openness and intrinsic motivation at work—qualities that facilitate engagement with uncertain, demanding tasks. This pattern is consistent with broaden-and-build theory, which holds that positive psychological states expand individuals’ cognitive and behavioral repertoires (Fredrickson, 2001).

The direct path from PWB to IWB was modest in practical magnitude, however, suggesting that psychological well-being may be insufficient on its own to explain innovation, and more specific psychological resources may also be relevant to the psychological conditions—particularly creative self-belief—through which innovative action becomes possible. This finding reinforces the study’s core argument that the association between well-being and innovation is not automatic and may operate partly through resource-specific indirect pathways, particularly creative self-efficacy.

### 5.2. The Belief-Based Pathway: Why Creative Self-Efficacy Matters for Innovation

The indirect pathway from PWB to IWB through CSE was statistically significant and practically meaningful. These findings indicate that creative self-efficacy represents a meaningful statistical pathway through which well-being was associated with innovative behavior. Employees who reported stronger psychological well-being also reported stronger beliefs in their own creative capability, and those with stronger creative self-beliefs also reported higher levels of innovative work behavior.

Social cognitive theory explains this pattern through the mechanisms of behavioral initiation, sustained effort, and persistence under difficulty (Bandura, 1997). In innovation contexts, these mechanisms matter especially because innovative behavior involves ambiguity, social risk, and the advocacy of unconventional ideas against possible organizational resistance. Employees who doubt their creative capability may remain behaviorally passive even when energized. Those with strong creative self-beliefs, by contrast, are more willing to challenge routines, take creative risks, and defend novel ideas—behaviors that ICT environments both require and reward.

These results align with and extend prior evidence identifying CSE as one of the strongest individual-level predictors of innovative behavior across organizational contexts (Newman et al., 2018; Tierney & Farmer, 2002; Ng & Lucianetti, 2016), and place that evidence within a digitally dynamic sector where creative confidence may be especially consequential.

### 5.3. The Energy-Based Pathway: Why Subjective Vitality Did Not Predict Innovation

The absence of a meaningful association between subjective vitality and innovative work behavior warrants closer interpretation, because vitality was nevertheless strongly associated with psychological well-being. Situating this pattern within prior evidence, comparable findings exist in which energetic or affective activation fails to translate fully into innovation-specific outcomes unless accompanied by additional agency-related resources. van Zyl et al. (2021) reported that the association between the energy-laden state of work engagement and innovative work behavior among ICT employees was weaker than the associations reported in prior non-ICT samples; Op den Kamp et al. (2020) found that proactive efforts to mobilize vitality benefited creative work performance only when coupled with complementary psychological resources such as self-insight; and Li et al. (2025) observed that, even in a lower-activation teaching context, the efficacy-based indirect pathway to innovative behavior accounted for a substantially larger share of the total indirect effect than the vitality-based pathway. These converging findings indicate that the present result may reflect a broader empirical pattern—energetic activation may not always be sufficient for innovation-specific outcomes when agency-related psychological resources are absent—rather than a sample-specific anomaly.

The contextual argument developed in this study permits an empirical evaluation of the two candidate explanations for this pattern. The range restriction account—whereby the structural characteristics of ICT work (compressed innovation cycles, continuous cognitive novelty, project-based urgency, and the intrinsic stimulation of working at the frontier of technological change; Tidd & Bessant, 2020) uniformly elevate baseline activation, normalize vitality, and narrow its variance—carries clear distributional implications. The descriptive statistics reported in this study do not bear these implications out: vitality exhibited the lowest mean, the most symmetric distribution, and the largest standard deviation among the study constructs, with no evidence of compressed upper-range responding. The observed distributional evidence therefore does not support the range-restriction account within the present sample. Critically, this strengthens rather than weakens the informativeness of the null finding: ample variance in vitality was available for an effect to emerge, and none did. The observed pattern is therefore more consistent with the resource-conversion account than with the range-restriction account. Although the present design does not test the conversion process directly, the observed pattern is consistent with this interpretation: psychological well-being was strongly associated with subjective vitality, yet vitality was not associated with innovative behavior, whereas creative self-efficacy was. Given the cross-sectional, self-report design, this interpretation should be read as a plausible explanation consistent with the data rather than as a mechanism demonstrated by them; a direct test would require designs that measure or manipulate the conversion process itself.

This evaluation carries broader theoretical implications. This pattern is unlikely to be explained by range restriction within the present sample and may instead reflect differences in the innovation relevance of generalized psychological energy and domain-specific efficacy beliefs. Whether range restriction dynamics nevertheless operate in other high-activation sectors, and whether the resource conversion account generalizes beyond ICT, are questions for cross-industry research spanning sectors with differing baseline activation levels.

One plausible interpretation is that vitality supports general engagement and persistence, whereas innovative work behavior also involves uncertainty, social risk, and confidence in one’s creative competence. Employees may therefore feel energized without acting innovatively when they lack creative confidence or perceive innovation-related behavior as risky. Creative self-efficacy may consequently serve as an important complement to psychological energy. This interpretation is consistent with the observed associations, but it was not directly tested by the cross-sectional design.

### 5.4. Theoretical Contributions

The principal contribution of this study lies in the direct comparison of two theoretically distinct indirect pathways within the same model. By examining subjective vitality and creative self-efficacy simultaneously, the study provides a clearer assessment of their relative associations with innovative work behavior. The results indicate that the belief-based pathway through creative self-efficacy was more closely associated with innovative behavior than the energy-based pathway through subjective vitality. This contribution should be understood as a focused extension of existing evidence rather than as a reconceptualization of the broader well-being–innovation literature.

The findings also illustrate the complementary relevance of self-determination theory and social cognitive theory. Psychological well-being was strongly associated with subjective vitality, consistent with the energetic perspective of self-determination theory, whereas creative self-efficacy was more closely associated with innovative work behavior, consistent with the emphasis of social cognitive theory on domain-specific efficacy beliefs. The resource-conversion account offers one plausible interpretation of this pattern, but the present design does not test the conversion process directly.

Finally, the study extends this comparison to an ICT-sector sample. However, ICT-specific characteristics were not directly measured; therefore, the findings do not establish that technological turbulence, digital work intensity, or AI-assisted work caused the observed difference between the two pathways. Future studies should test these contextual characteristics directly as potential moderators.

### 5.5. Limitations and Future Research Directions

Several limitations should be acknowledged. First, the cross-sectional design limits causal inference and prevents direct observation of the temporal ordering among variables (Stone-Romero & Rosopa, 2008). The findings should therefore be interpreted as reflecting associations rather than causal pathways. Future research should employ longitudinal, time-lagged, or experimental designs to examine whether PWB prospectively predicts CSE and subsequently IWB.

Second, the study relied on a single-source self-report design, which, despite the procedural and statistical CMV remedies applied, remains a methodological limitation. Future research may benefit from multi-source data collection—for instance, supervisor ratings of innovative work behavior alongside employee self-reports of psychological variables. Because all constructs were measured from the same source at a single point in time, the observed associations may be partially inflated by shared method variance despite the procedural remedies, full-collinearity assessment, and Harman’s test reported above, and the mediation estimates should accordingly be interpreted as statistical rather than temporal decompositions of effects.

Third, the convenience sample was drawn exclusively from ICT employees in Türkiye, which limits the generalizability of findings across sectors, national cultures, and organizational contexts with differing innovation demands. Comparative cross-cultural or cross-sector studies would substantially strengthen the external validity of these findings. Moreover, recruitment through LinkedIn and organizational e-mail lists may over-represent more highly educated and professionally networked employees relative to the broader ICT workforce, introducing potential selection bias; future studies should consider organization-based or stratified sampling designs.

Importantly, although the ICT sector provides the contextual frame for the study, ICT-specific work characteristics were not directly measured. The model does not include measured indicators of ICT-specific characteristics—such as perceived technological turbulence, digital work intensity, or the degree of AI-assisted work—so the contextual argument operates at the level of sample framing rather than measured variables; future studies should incorporate such indicators as moderators to test the contextual claims directly. A further sample-related limitation concerns gender composition: the sample was predominantly male (83.4%). Although this distribution broadly mirrors the gender structure of the Turkish ICT workforce, it constrains the generalizability of the findings to female ICT employees. A sensitivity analysis reported in this study indicated that gender was not significantly associated with innovative work behavior and that the hypothesized path coefficients remained substantively unchanged; however, the female subsample (n = 77) was too small to support a reliably powered multi-group comparison of the path coefficients, and future research should therefore test the model in more gender-balanced samples.

Fourth, the model examined four constructs and did not incorporate additional psychological or contextual variables that may moderate or mediate these relationships. The current model is psychologically clean and theoretically parsimonious—which is a strength for conceptual clarity and model parsimony—but it does not capture the conditional complexity that may characterize these relationships in organizational settings. Future studies should investigate whether the strength of the CSE–IWB and SV–IWB pathways is contingent on contextual boundary conditions. Candidate moderators include: (a) psychological safety climate, which may amplify CSE’s effect on IWB by reducing the perceived social risk of creative action; (b) leadership style (e.g., transformational or empowering leadership), which may strengthen or weaken the translation of CSE into innovative behavior; (c) organizational innovation climate, which may differentially facilitate CSE-driven versus vitality-driven innovation; (d) job autonomy, which may interact with creative self-efficacy to produce especially strong innovation effects; and (e) uncertainty avoidance at both the individual and cultural level, which may moderate how confidently employees translate creative beliefs into visible innovative behavior. Incorporating such boundary conditions in future models would substantially deepen the theoretical sophistication of this line of inquiry and address one of the more persistent critiques of linear mediation models in organizational psychology.

Finally, future research may benefit from examining innovative work behavior as a multidimensional construct with distinct phases—idea generation, idea promotion, and idea implementation—as different psychological resources may be differentially relevant across these stages. For instance, subjective vitality may contribute more meaningfully to implementation and persistence-related stages, while creative self-efficacy may be most critical during idea generation and advocacy. In particular, the non-significant role of subjective vitality warrants targeted follow-up through three specific designs: cross-industry comparative studies sampling sectors with contrasting baseline activation levels (e.g., ICT versus public administration or traditional manufacturing) to test the range-restriction account directly; longitudinal designs tracking whether within-person changes in vitality precede changes in innovative behavior; and experimental or intervention studies that manipulate energization inputs and creative-efficacy inputs independently to disentangle their causal contributions.

### 5.6. Practical Implications

The findings carry several implications for organizations in the ICT sector. Given the cross-sectional design and the sector-specific sample, these implications should be read as ICT-focused and associational rather than causal, and their generalization to other industries warrants caution. First, investing in employees’ psychological well-being should be understood not only as an ethical responsibility but as a strategically important organizational priority associated with innovation-oriented functioning. Organizations may therefore benefit from implementing practices that support eudaimonic well-being specifically—including meaningful work design, autonomy-supportive management, and clarity of organizational purpose—rather than relying exclusively on hedonic wellness programs.

Second, and most directly, the central role of creative self-efficacy in the PWB–IWB pathway suggests that organizations seeking to strengthen innovation capacity may benefit from cultivating employees’ beliefs in their own creative capability. Developmental practices such as mastery-oriented feedback, creativity training, visible recognition of innovative contributions, and structured reflection on past creative successes may strengthen creative self-efficacy and increase employees’ willingness to engage in innovation-related behavior. Psychologically safe environments that normalize creative failure and experimentation are likely to be particularly important, as they reduce the perceived social risk of innovative action and reinforce employees’ beliefs that their creative contributions are valued.

In ICT settings specifically, creative self-efficacy can be cultivated through practices native to software and technology work: structured internal hackathons and innovation sprints with explicit organizational follow-through on selected ideas; mastery-oriented code and design reviews that frame feedback around capability growth rather than error detection; planned rotation of employees into proof-of-concept and R&D tasks that provide graduated mastery experiences; internal tech talks and demo days that give visible recognition to novel contributions; blameless post-mortems that institutionalize learning from failed experiments; and mentoring by senior technical staff who explicitly model and verbally affirm creative problem-solving—verbal persuasion and vicarious experience being core sources of efficacy beliefs in social cognitive theory (Bandura, 1997).

Third, the limited contribution of subjective vitality to innovative outcomes in this ICT sample carries a strategic implication for digital transformation management. Organizations undergoing rapid technological change frequently invest in employee engagement and energization programs—high-vitality cultures, agile work environments, gamified task structures—on the assumption that more energized employees will naturally become more innovative. The present findings caution against this assumption, and on grounds more precise than saturation: in this sample, vitality was neither uniformly high nor lacking in variance, yet it still was not associated with innovative behavior—suggesting that, within the present sample, the more relevant differentiating factor may have been creative confidence rather than employees’ general level of psychological energy. Rather than asking only whether their workforce is sufficiently energized, organizations should therefore diagnose where their innovation bottleneck lies.

Brief periodic pulse surveys measuring both vitality and creative self-efficacy with validated items can establish the distribution of each resource across teams. Where vitality is low or highly uneven (for example, teams operating under sustained crunch conditions), energization- and recovery-oriented interventions retain clear value. Where vitality is adequate but creative self-efficacy is low or unevenly distributed—the pattern most consistent with the present findings—additional energization spending may have limited innovation-related benefits unless complemented by belief-building practices, and resources may be more usefully directed toward complementary belief-building practices.

Organizations may therefore benefit from investing in practices that cultivate creative self-efficacy—the belief-based resource that, in the present findings, was most strongly associated with translating psychological readiness into innovative action. Practically, this suggests a reorientation of innovation-focused HR practices toward mastery-building experiences: structured creative challenges, visible recognition of novel contributions, psychologically safe failure debriefs, and leadership behaviors that explicitly affirm employees’ creative competence. These investments target the belief-based mechanism that the present findings identify as an important psychological correlate of employee innovation in digitally dynamic organizational contexts.

These implications acquire additional force in AI-enabled workplaces. As AI assistants absorb a growing share of executable and analytical work, the marginal human contribution shifts toward creative direction, problem framing, and judgment (Raisch & Krakowski, 2021), and emerging evidence indicates that the innovation benefits of AI assistance are transmitted through employees’ creative self-efficacy (Yin et al., 2024; Jia et al., 2024). In such environments, the belief-building practices identified above may represent an important lever for realizing the potential innovation benefits of AI investment: employees with lower creative self-efficacy may be less likely to translate AI-augmented capacity into innovative action.

## 6. Conclusions

The central finding of this study is that the indirect association between psychological well-being and innovative work behavior differed across the two psychological resources examined. Among ICT employees, creative self-efficacy represented a meaningful indirect pathway, whereas subjective vitality did not, despite its strong association with psychological well-being. This asymmetry supports a more differentiated understanding of the psychological resources associated with innovative behavior.

In the present sample, subjective vitality was neither uniformly elevated nor restricted in variance, but it was not meaningfully associated with innovative work behavior. Creative self-efficacy, by contrast, was more closely associated with innovation-related action. This pattern is consistent with the possibility that domain-specific creative confidence may be more relevant to innovative behavior than generalized psychological energy, although longitudinal or experimental research is required to examine the underlying process directly.

As organizations navigate digital transformation and intensifying innovation demands, the psychological conditions through which well-being is associated with creative action deserve closer and more differentiated attention. The present findings suggest that not all well-being resources are equally innovation-relevant, and that creative self-efficacy may occupy a particularly important position in the psychological architecture of employee innovation. Future inquiry into the contextual conditions that cultivate or constrain such beliefs—across sectors, leadership systems, and cultural settings—would substantially deepen the practical and theoretical reach of this line of research.

## Figures and Tables

**Table 1:** Demographics of the Participants (N = 463).

Demographics	Options	f	%	Cumulative %
Age	18–25	59	12.7	12.7
	26–33	207	44.7	57.5
	34–41	130	28.1	85.5
	42–49	49	10.6	96.1
	50–57	16	3.5	99.6
	58 and above	2	0.4	100.0
Gender	Female	77	16.6	16.6
	Male	386	83.4	100.0
Marital Status	Married	252	54.4	54.4
	Single	211	45.6	100.0
Education Level	Highschool	15	3.2	3.2
	Associate Degree	75	16.2	19.4
	Bachelor Degree	247	53.3	72.8
	Master Degree	116	25.1	97.8
	PhD	10	2.2	100.0
Tenure in Current Position	0–2 Years	194	41.9	41.9
	3–5 Years	108	23.3	65.2
	6–8 Years	63	13.6	78.8
	9+ Years	98	21.2	100.0
Total Work Experience	0–2 Years	47	10.2	10.2
	3–5 Years	82	17.7	27.9
	6–8 Years	72	15.6	43.4
	9+ Years	262	56.6	100.0

**Table 2:** Measurement Model: Factor Loadings, Reliability, and Convergent Validity.

Construct/Item	Factor Loadings	Cronbach α	CR	AVE
Psychological Well-Being (PWB)		0.870	0.898	0.523
PWB01	0.716			
PWB02	0.734			
PWB03	0.706			
PWB04	0.725			
PWB05	0.753			
PWB06	0.751			
PWB07	0.680			
PWB08	0.720			
Creative Self-Efficacy (CSE)		0.937	0.948	0.694
CSE01	0.741			
CSE02	0.839			
CSE03	0.864			
CSE04	0.848			
CSE05	0.850			
CSE06	0.843			
CSE07	0.818			
CSE08	0.852			
Innovative Work Behavior (IWB)		0.876	0.908	0.625
IWB01	0.835			
IWB02	0.843			
IWB03	0.559			
IWB04	0.815			
IWB05	0.832			
IWB06	0.822			
Subjective Vitality (SV)		0.879	0.908	0.593
SV01	0.856			
SV02	0.497			
SV03	0.612			
SV04	0.851			
SV05	0.790			
SV06	0.786			
SV07	0.912			

**Table 3:** Discriminant Validity (HTMT).

Construct	PWB	CSE	IWB	SV
Psychological Well-Being (PWB)	—			
Creative Self-Efficacy (CSE)	0.559	—		
Innovative Work Behavior (IWB)	0.591	0.696	—	
Subjective Vitality (SV)	0.729	0.427	0.442	—

**Table 4:** Collinearity Statistics: Variance Inflation Factor (VIF) Values.

Predictor	VIF	CMV Assessment
Psychological Well-Being (PWB)	2.016	No concern (<3.3)
Creative Self-Efficacy (CSE)	1.365	No concern (<3.3)
Subjective Vitality (SV)	1.767	No concern (<3.3)

**Table 5:** Explained Variance (R^2^) and Predictive Relevance (Q^2^).

Predictor	R^2^	R^2^ Adjusted	Q^2^
Creative Self-Efficacy (CSE)	0.261	0.259	0.173
Innovative Work Behavior (IWB)	0.462	0.458	0.280
Subjective Vitality (SV)	0.429	0.428	0.249

**Table 6:** Path Coefficients.

Path	β	SE	t	*p*	95% CI
PWB → CSE	0.511	0.056	9.130	<0.001	[0.396, 0.615]
PWB → SV	0.655	0.030	21.509	<0.001	[0.585, 0.708]
PWB → IWB	0.225	0.056	3.986	<0.001	[0.113, 0.331]
CSE → IWB	0.504	0.062	8.155	<0.001	[0.366, 0.611]
SV → IWB	0.055	0.062	0.894	0.371	[−0.055, 0.188]
PWB → CSE → IWB	0.258	0.046	5.568	<0.001	[0.170, 0.349]
PWB → SV → IWB	0.036	0.041	0.883	0.377	[−0.037, 0.122]

## Data Availability

The data presented in this study are available from the corresponding author upon reasonable request. The data are not publicly available because public sharing was not covered by the informed consent obtained from the participants. Furthermore, access to the data is restricted to protect participant confidentiality and comply with ethical requirements.

## Abbreviations

The following abbreviations are used in this manuscript:

PWB	Psychological well-being
IWB	Innovative work behavior
SV	Subjective vitality
CSE	Creative self-efficacy
CR	Composite reliability
AVE	Average variance extracted
HTMT	Heterotrait–monotrait ratio
VIF	Variance inflation factor
PLS-SEM	Partial least squares structural equation modeling
